# Oral Immunotherapy (OIT): A Personalized Medicine

**DOI:** 10.3390/medicina55100684

**Published:** 2019-10-13

**Authors:** Francesca Mori, Simona Barni, Giulia Liccioli, Elio Novembre

**Affiliations:** Allergy Unit, Department of Pediatrics, Anna Meyer Children’s University Hospital, 50139 Florence, Italy; s.barni@meyer.it (S.B.); giulialiccioli@gmail.com (G.L.); e.novembre@meyer.it (E.N.)

**Keywords:** children, desensitization, egg allergy, food allergy, milk allergy, oral immunotherapy, peanuts allergy, tree nuts allergy, wheat allergy

## Abstract

Oral Immunotherapy (OIT), a promising allergen-specific approach in the management of Food Allergies (FA), is based on the administration of increasing doses of the culprit food until reaching a maintenance dose. Each step should be adapted to the patient, and OIT should be considered an individualized treatment. Recent studies focused on the standardization and identification of novel biomarkers in order to correlate endotypes with phenotypes in the field of FA.

## 1. Introduction

Oral Immunotherapy (OIT), a promising allergen-specific approach in the management of Food Allergies (FA) [1], is based on the administration of increasing doses of the culprit food until reaching a maintenance dose, after which a regular intake of the specific food allergen is mandatory for “desensitizing” the patients to prevent exposure triggering an allergic reaction. While the main goal of the research is to induce sustained unresponsiveness or tolerance, OIT serves to introduce allergenic food into the normal diet, or in case of high-risk individuals, to introduce low doses in order to prevent severe reactions after accidental exposure [2].

In this regard, some researchers believe it is clinically and psychologically significant if a small amount of tolerance exists, enabling patients to tolerate accidental ingestion of the culprit food. Normally, FA is not just about tolerance or non-tolerance of the offending food. Recently, different phenotypes have been described, for example, patients who tolerate the allergenic food heated but are not able to tolerate the whole food uncooked. For this reason, OIT introduces new concepts such as the eliciting dose, the “matrix effect” (i.e., the complex interaction with other proteins, fats, and carbohydrates in the food matrix able to change protein allergenicity), and the way of processing foods. As a result, there is huge diversity among studies on the target food and the vehicle substance employed for OIT (i.e., some foods available commercially are used in their natural forms, while others use processed foods like defatted peanuts or dehydrated egg white), so the comparison among them could be difficult. Consequently, in the US, the Food and Drug Administration (FDA) has imposed standardization in terms of safety and quantification/identification of allergenic proteins. In fact, pharmaceutical-grade peanut flour is currently being subjected to phase 3 trials (AImmune) and is expected to be widely available in the coming years [3].

Another standardized drug for peanut OIT, called AR101, was studied in a phase 3 clinical trial that enrolled 551 patients with peanut allergy aged from 4 to 55 years [4].

Nonetheless, OIT should be considered an individualized treatment; hence its standardization in a general protocol represents a challenge for investigators and clinicians alike. Each step must be adapted to the patients’ specific situation (i.e., infections, gastrointestinal disorders, drug assumption, exercise, adverse reactions during treatment). It is strongly recommended to conduct an oral food challenge (OFC) in order to establish the lowest reaction eliciting dose. Moreover, OFC could be used to assess the efficacy of desensitization while undergoing therapy, or functional tolerance when not on therapy and on a limited diet for a period of at least four weeks [5].

The initial doses for patients undergoing OIT are sufficiently low to avoid reactions and could be individualized or established for the whole study population [6,7].

Generally, the first step of OIT includes an escalation phase starting from micrograms of allergenic proteins and reaching the range of several milligrams in one to two days. The building up phase includes an increment of the dose twice or once a week until reaching a maintenance dose, or there is the onset of dose-limiting symptoms in the hospital setting under the supervision of healthcare professionals. This phase can last for varying amounts of time: from flash protocols (one week) to slow protocols (>6 months).

Low dose food challenges should be used in high-risk patients, while the more rapid introduction of an allergenic food could be performed in low-risk patients [8].

Individualized protocols may be recommended in patients with reactions to medium-high doses of the food in the challenge tests, thus making it possible to shorten the build-up phase, with savings in healthcare resources and greater comfort for the patient. However, the build-up starting dose with respect to the threshold dose has yet to be established (e.g., a cumulative dose of half a boiled egg or 50 mL of cow’s milk could be considered as the medium and high-tolerance thresholds) [9].

Moreover, enormous differences exist among studies in relation to the target maintenance dose range between 300 to 400 mg of allergenic proteins. In several studies, very high maintenance doses were administered (e.g., 4000 mg of peanut, equal to about 17 peanuts); however, the intake of high amounts of allergenic food is not clinically necessary and is frequently associated with a fear of reactions, and therefore, difficult to maintain because of aversion to the implicated food [10].

The target dose for milk OIT is usually 200–250 mL, even though 15 mL of milk may be considered the final milk dose in high-risk patients since it offers protection against minor accidental exposure and helps increase tolerance over time. An amount of 300 mg of powdered egg-white protein or the equivalent is considered safe for avoiding reactions deriving from traces, cross-contamination, or labeling errors [11].

In addition, the duration of the maintenance phase may be variable, as it could be prolonged for months to years and is characterized by the daily or at least biweekly administration of the offending food at home. Factors conditioning the duration of the maintenance phase are patient’s and families’ compliance and consistency, individual immunological response to an allergen, type of food and grade of allergenicity, the occurrence of an adverse reaction, etc.

Nevertheless, the extending of the maintenance phase can give rise to treatment compliance problems. In fact, drop-outs have been reported in about 60% of patients subjected to 3–5 years on cow’s milk and peanut OIT [12,13].

Moreover, it is not known what the outcome will be or how much time is required to reach permanent tolerance of the offending food. In the study on egg OIT, Jones et al. reported how tolerance is enhanced with the duration of OIT. The sustained unresponsiveness increases from 27.5% after two years of OIT, to 50% after four years of egg OIT [14].

With regard to the safety of OIT, over the last three years, major concerns have been examined in systematic reviews and meta-analyses, and it has been found that up to 91.5% of adverse reactions are observed during OIT in all patients treated and in 16% of the administered doses [15,16].

The majority of adverse reactions reported, which were mild and self-limiting, included itchiness of the mouth and lips, facial and generalized urticaria and erythema, abdominal symptoms, rhino-conjunctivitis, mild laryngeal spasms, and mild wheezing [17,18].

Severe anaphylactic reactions have been reported, and in the literature, the controlled clinical trials found that the intramuscular administration of epinephrine was necessary in 6.7% to 30.8% of all patients subjected to milk OIT, and in 20% of those subjected to egg OIT or peanut OIT [19,20].

Despite the fact that life-threatening reactions have been observed in asthmatic teenagers with poor compliance [19,20], several studies show that even though mild adverse reactions are quite common with OIT, they tend to become less frequent and less severe over time [14].

While adverse reactions normally occur with dose escalation, they are also possible during maintenance therapy with doses that were previously well-tolerated, due to being unpredictable and at times triggered by cofactors like infections, exercise, and anxiety [21,22].

In particular, adverse reactions could be the cause of patients withdrawing in 3–20% of cases of milk OIT and 0–36% of cases of egg OIT [10,23].

In addition, most withdrawals are due to the occurrence of gastrointestinal symptoms and not to anaphylaxis, with reports of eosinophilic esophagitis (EoE) in 2.7% of patients subjected to milk, peanut, egg and wheat OIT [7,12,22,24,25]. In this review, we focused, in particular, on milk, egg, and peanut because they are the most frequently implicated in food allergy in children.

## 2. Quality of Life (QoL)

The variable impact that OIT can have on the quality of Life (QoL) probably depends on the QoL at baseline. There was great improvement in patients with impaired QoL at the beginning, whereas deterioration was observed in those with acceptable QoL at baseline [26]. Moreover, the number of doses tolerated by patients seems to be inversely associated with QoL. The more allergic patients who had frequent reactions during the first phase of OIT, even with a few tolerated doses, failed to show any improvement in their QoL, compared to a restricted diet. Conversely, for those children who completed the OIT protocols, an improved QoL was reported [27].

## 3. Immunologic Changes with OIT

During OIT, the T helper (Th)2/Th1 ratio is reduced and the T regulatory effector cells increase with the production of Interleukin 10 (IL-10) by the APCs (antigen-presenting cells) and the activation of immune cells which together with Transforming growth factor-beta (TGF-β) induce production of Immunoglobulin G4 (IgG4) and IgA. It is assumed that food-specific IgG4 during OIT could have an antigen-neutralizing effect and decreased basophil and mast cell responsiveness, with the suppression of IgE production. In allergen binding, both IgG4 and IgA compete with IgE, decreasing the allergen capture by basophils. This leads to a reduction in the amount of specific IgE, but also in the diversity of epitope recognition and altered affinity of IgE for antigens.

Decreased allergen-induced skin prick test (SPT) and basophil activation during the first few months of immunotherapy have been observed in OIT studies. However, a typical early increase in food-specific IgE levels has been demonstrated during the initial months of OIT. After 6 to 12 months of OIT, a transition has been observed from a Th2 predominant cytokine signature to a Th1-associated pattern, while immune suppression by T regulatory (T reg) cells and clonal anergy occur later during OIT. Syed et al. reported an increase in the function of antigen-specific CD4 + CD25 + Foxp3 + T Treg cells following OIT, corroborating the theory of active suppression of the immune response by food allergens [24,25,28,29,30,31,32,33].

## 4. Sustained Unresponsiveness

Immunologic changes occurring during OIT-treatment seem to be temporary, revealing interindividual variability in immune suppression and clinical response. In egg OIT, from 71% to 90% of maintenance-phase patients retain desensitization after 1–6 years of follow-up [34,35,36,37,38]. In the literature, the length of follow-up reported in milk OIT ranges from 3 to 5.8 years. Desensitization to a full serving dose of cow milk (CM) equal to 200 mL is maintained in a range of between 31 and 100% of subjects [12,39,40,41]. The avoidance period before retesting tolerance has been described as being as long as 1–4 months. One study on peanut allergy reported that 50% of patients (3/6 individuals) who passed an initial challenge test after a three-month avoidance diet following successful completion of OIT, had a positive second challenge test after the avoidance period was prolonged for a further three months [32]. Today, milk and peanut OIT are the most widely studied (Table 1 and Table 2). There are very few publications on the egg (Table 3), other tree nuts (Table 4), wheat (Table 5), or fish OIT.

## 5. Multiple Food OIT

A limit of OIT is represented by the fact that it is an allergen-specific approach, and most studies have been carried out on a single allergen, even though a great number of patients are sensitive to multiple allergens. For this reason, recent studies involving multiple foods have been described [89].

Moreover, in the case of tree nut allergy, cross-desensitization has been reported with the ingestion of only one type of nut thanks to the relevant cross-reactivity among tree nuts [83].

## 6. Desensitization Efficacy

A recent systematic meta-analysis of 31 clinical trials on food allergy [90] demonstrated the efficacy of desensitization, which entails an increase in the reaction threshold calculated as the food dose tolerated by the patient.

## 7. Personalized Medicine

It might be helpful to identify biomarkers associated with safe and successful OIT in order to select suitable subjects who are not expected to have reactions to OIT, screening out subjects in whom OIT could give rise to unnecessary risks.

While it is likely that the outcome of OIT depends on numerous factors, several individual characteristics could have a predictive value.

There is evidence that the following patients are at high risk for failing OIT “desensitization”:

1. with IgE binding to a boarder diversity of peptides;
2. with high IgE-binding intensity to allergens:
3. with the highest level of serum- specific IgE or the largest skin test response;
4. with more severe reactions at low doses;
5. with more severe asthma;
6. with persistent allergy (desensitization could prove to be more effective in small children, suggesting that it is easier to achieve immune modulation when started at an early age) [10,43,58,59,91,92].

There is evidence indicating that the following patients as more likely to successfully complete OIT “desensitization”, namely, those:

1. who are able to tolerate some form of allergen, e.g., patients eating cooked milk or cooked egg, may outgrow the overall allergy sooner [93,94,95];
2. who show a reduced skin prick test wheal size and an increase in specific IgG4-blocking antibodies after OIT to cow’s milk, egg, and peanut [14,24], with the latter possibly being a biomarker for sustained unresponsiveness [91];
3. who show a tendency towards a decrease in the specific IgE levels.

## 8. Conclusions

Clinical and immunopathological studies on FA-OIT focus on novel biomarkers and therapies in order to correlate FA endotypes with clinical phenotypes and propose the best-personalized treatment for each patient with FA.

Moreover, more research is necessary for understanding whether a longer course of OIT could increase tolerance rates and whether OIT only accelerates desensitization in subjects who would, in any case, progress towards natural tolerance without any intervention.

## Figures and Tables

**Table 1:** Milk OIT studies.

Reference, Year	Design	Sample Size (n)	Subject Age (yrs)	Maintenance Dose	Duration	Conclusions
**Meglio P. et al., 2004** [42]	open-label	21	6–10	200 mL	6 mon	72% achieved desensitization to 200 mL of cow’s milk daily
**Longo G. et al., 2008** [43]	randomized open-label	30	5–17	150 mL	10-day rush escalation, 1 yr maintenance	36% completely tolerant (≥150 mL) and 54% partially tolerant (5–150 mL)
**Skripak JM. et al., 2008** [11]	randomized, placebo-controlled	13	6–17	500 mg milk protein	23 wk	Median milk challenge threshold increased from 40 mg at baseline to 5140 mg after OIT
**Narisety SD. et al., 2009** [44]	open-label (follow-up)	13	6–16	500–4000 mg milk protein	3–17 mo	Ongoing milk intake demonstrated tolerance from 1000 to 16,000 mg (median, 7000) with 33% tolerating 16,000 mg on OFC
**Pajno GB. et al., 2010** [45]	randomized, placebo-controlled	15	4–10	200 mL	18 wk	67% tolerant to 200 mL cow’s milk
**Martorell A. et al., 2011** [46]	randomized, placebo-controlled	30	2–3	200 mL	1 yrs	90% showing complete desensitization
**Keet CA. et al., 2012** [47]	randomized, placebo-controlled	20 for OIT	6–17	1000–2000 mg	60 wk	70% of patients receiving OIT passed an 8 g OFC.; only 40% passed OFC when treatment was discontinued for 6 wk
**Goldberg M. et al., 2015** [48]	open	14	6.5–12.7	1.3 g of BM protein	12 mo	Only 3 (21%) of 14 patients tolerated the 1.3 g/d BM dose. Patients who successfully reached maintenance had decreased milk-specific IgE reactivity.
**Takahashi M. et al., 2016** [49]	open	31 (48 tot, 31 OIT, 17 controls)	5–17	200 mL of microwave heated cow’s milk every day (fresh cow milk was warmed in a microwave oven at 550 W for100 s)	12 mo	No children in the untreated group did not pass an open food challenge to CM. Of the 31 children in the OIT group, 14 (*p* = 0.002) achieved desensitization, and eight (*p* = 0.036) achieved two-weeks-SU to CM at 1 year from the start of OIT. Two years after the start of OIT, both the rate of desensitization and the rate of the two-week-SU in the OIT group significantly increased compared with the rates at one year (*p* = 0.025 and *p* = 0.008, respectively).
**Ebrahimi M. et al., 2017** [50]	open	14	3.5–7	200 to 250 mL of cow’s milk each day for 90 days.	90 days	The median of the difference of the wheel diameter with the control, decreased from 10 to 6 mm. After the OIT, the sIgE level of cow’s milk proteins and casein decreased from 39.30 to 10.40 and 7.72 to 2.83 (KU/L), respectively. The study doesn’t show data of sustained responsiveness in the follow-up.
**Amat F. et al., 2017** [51]	randomized	43 (18 high-risk arm, 23 low-risk arm)	3–10	“low-risk arm”: from extensively heated baked milk to the half-heated baked milk and then raw milk until 2720 mg of milk protein per day. “high-risk arm”: immediately raw milk	9 mo	Fifteen children (36.6%) were classified as responders, 11 (26.8%) were partial responders, with an average gain in threshold of tolerance of 697 mg [27.2–2550], and 15 children (36.6%) remained non-responders. The study doesn’t evaluate sustained unresponsiveness to milk proteins
**Efron A. et al., 2018** [52]	retrospective, case-control	43 (110 tot, 43 OIT, 67 controls)	1–4	First OFC—cookie containing ~1 g milk protein heated in frying and baking. Second OFC—pancake containing ~1 g milk protein heated in frying. Third OFC—toast containing ~4 g cheese proteins (mostly casein). Fourth OFC—yogurt containing ~4 gr of unheated cheese proteins.	12–18 mo (3 mo each product)	At last follow-up, 86% of treated children were tolerant to unheated milk proteins vs. 52% of controls (*p* = 0.003).
**Inuo C. et al., 2018** [53]	randomized, double-blind, controlled	25 (13 pHF-pHF, 12 eHF-pHF)	1–9	two double-blind groups: a partially hydrolyzed cow’s milk protein-based formula (pHF)-pHF group and an extensively hydrolyzed cow’s milk protein-based formula (eHF)-pHF group	16 wk	There was a significant increase in the threshold in the pHF-pHF group (*p* = 0.048), but not in the eHFpHF group (*p* = 0.23). Among the participants with a severe allergy, whose baseline thresholds were <4 mL, there was a significant change in thresholds between baseline and at the end of the trial in the pHF-pHF group (*p* = 0.023).
**Mota I. et al., 2018** [54]	prospective	42	2–18	200 mL	36 mo	During the maintenance phase, 92% maintained diet without restrictions including daily ingestion of 200 mL of CM (36 of 39 adherent patients). Overall, 93% were adherent patients (39 of 42), since they keep daily ingestion of 200-mL CM.
**Kauppila T. et al., 2019** [55]	open	180 (296 OIT, 64 controls)	5–17	200 mL	11 yrs of follow-up	Out of the initial study group, 244/296 (83%) patients participated in the long-term follow-up. Among these patients, 136/244 (56%) consumed ≥2 dL of milk daily. The median follow-up time was 6.5 years. Of the recorded markers and clinical factors, the baseline milk sIgE level was most associated with maintaining milk OIT (*p* < 0.001).
**De Schryver S. et al., 2019** [56]	open	26 (52 tot, 26 OIT and 26 controls)	6–18	200 mL	1 mo	Among the 26 children randomized to OIT, 18 were defined as desensitized to milk. The difference in the percentage of milk-desensitized children between the groups attributed to the OIT is 69.2%
**Berti I. et al., 2019** [57]	open	68	3–11 mo	up dosing until 150 mL	3.5–16 mo	Sixty-six infants (97%) reached the target of the protocol

Legend: y: years; mo: months; n: number; OFC: oral food challenge; OIT: oral immunotherapy; tot: total; wk: weeks; BM: baked milk; SU: sustained unresponsiveness.

**Table 2:** Peanut OIT studies.

Reference, Year	Design	Sample Size (n)	Subject Age (yrs)	Maintenance Dose (mg)	Duration	Conclusions
**Jones SM. et al., 2009** [24]	open-label	29	1–16	1800	36 mo	93% passed 3.9 g peanut OFC
**Blumchen K. et al., 2010** [58]	randomized, open-label	23	3–14	500	7-day rush escalation, 8 wk maintenance	64% reached their maintenance dose of 500 mg peanut
**Varshney P. et al., 2011** [25]	randomized, placebo-controlled	19	3–11	2000	48 wk	84% passed 5000 mg peanut OFC
**Anagnostou K. et al., 2011** [59]	open-label	22	4–18	800	32 wk	64% tolerated 6.6 g OFC
**Anagnostou K. et al., 2014** [60]	randomized, placebo-controlled	39	7–16	800	26 wk	62% tolerated 1400 mg challenge
**Vickery BP. et al., 2014** [10]	open-label	24	1–16	≤ 4000	≤ 5 y	1 mo after OIT stopped, 50% achieved sustained unresponsiveness to 5000 mg OFC
**Narisety SD. et al., 2015** [6]	randomized, placebo-controlled	16	7–13	2000	12 mo	Significantly greater increase in OFC threshold in OIT vs. SLIT, low rate of sustained unresponsiveness
**Kukkonen K. et al., 2017** [61]	double-blind, placebo-controlled	39 (60 tot, 39 OIT and 21 controls)	6–18	100-2000	8 mo	85% of patients passed the build-up phase, and 67% tolerated 5 g of peanuts during the post-treatment challenge
**Vickery B. et al., 2017** [62]	double-blind, placebo-controlled	40 (40 OIT and 154 controls)	9–36 mo	300-3000	29 mo	overall 78% of subjects receiving E-OIT demonstrated sustained unresponsiveness to peanut four weeks after stopping E-OIT and reintroduced peanut into the diet
**Bird JA. et al., 2018** [63]	double-blind, placebo-controlled	29 (tot 55, 29 OIT and 26 controls)	4–26	300	20-34 wk	79% and 62% AR101 subjects tolerated > 443 mg and 1043 mg respectively, versus 5 of 26 (19%) and 0 of 26 (0%) placebo subjects (both *p* < 0.0001)
**PALISADE group, 2018** [4]	double-blind, placebo-controlled	372 (496 tot, 372 OIT and 124 controls)	4–17	300	24 wk	250 of 372 participants (67.2%) who received active treatment, as compared with 5 of 124 participants (4.0%) who received placebo, were able to ingest a dose of 600 mg or more of peanut protein, without dose-limiting symptoms, at the exit food challenge
**Nachshon L. et al., 2018** [64]	prospective	139 (145 tot, 139 < 18 y)	4–18	1200 or 3000	6 mo	Of the 145 patients treated, 113 (77.9%) were fully desensitized to 3000 mg of peanut protein, 20 (13.8%) patients were partially desensitized to 300-2400 mg, and 12 patients (8.3%) failed. 63/64 patients (98.4%) consuming 1200 mg maintenance dose were successfully re-challenged to 3000 mg. All patients in the high dose group (3000 mg) who continued regular consumption and arrived for follow-up (n = 22) passed a challenge to 3000 mg.
**Nagakura K. et al., 2018 PAI** [65]	prospective, open-label	24 (24 OIT, 10 controls)	5–18	133	12 mo	16 children (67%) passed the 133-mg OFC, and 14 (58%) passed the 795-mg OFC. Only 1 child (10%) in the historical control group passed the 133-mg OFC (*p* = 0.006). Ultimately, eight children (33%) in the OIT group achieved sustained unresponsiveness
**Nagakura K. et al., 2018** [66]	double-blind, placebo-controlled	22 (22 OIT, 11 controls)	5–18	795	2 y	15/22 patients (68.1%) in the OIT group achieved sustained unresponsiveness, whereas only 2 (18.1%) in the control group passed the second OFC
**Anvari S. et al., 2018** [67]	double-blind, placebo-controlled	15	5–16	3900	3 mo	OIT participants who underwent dose variations on the unexpired lots of peanut flour were able to successfully tolerate the 100% dose increase, following a two-week tolerance of a 50% dose reduction on an unexpired lot of peanut flour
**Zhong Y. et al., 2018** [68]	open-label	7 (9 total, 7 completed protocol)	8–14	3000	12 mo	Of the seven who completed OIT, six tolerated 6000 mg of peanut protein at the first OFC at six months of maintenance phase; the last patient was afraid of consuming more than 3000 mg of peanut protein but passed the challenge with 3000 mg. After 12 months of maintenance therapy, only 3 of the 7 subjects consented to 4 weeks of abstinence. Of these, only 1 passed the challenge with 6000 mg of peanut protein.
**Fauquert JL. et al., 2018** [69]	double-blind, placebo-controlled	21 (30 tot, 21 OIT and nine controls)	12–18	400 IN CAPSULES	24 wk	Unresponsiveness to 400 mg of peanut protein was achieved in 17/21 peanut group patients (two patients withdrew) and 1/9 in the placebo group
**Blumchen K. et al., 2019** [70]	double-blind, placebo-controlled	31 (62 tot, 31 OIT and 31 controls)	3–17	125–250	16 mo	Twenty-three of 31 (74.2%) children of the active group tolerated at least 300 mg peanut protein at final food challenge compared with 5 of 31 (16.1%) in the placebo group (*p* < 0.001). Thirteen of 31 (41.9%) children of the active versus 1 of 31 (3.2%) of the placebo group tolerated the highest dose of 4.5 g peanut protein at final OFC (*p* < 0.001)
**Wasserman RL. et al., 2019** [71]	retrospective record	270	4–18	3000	36 mo	All patients who reached the 3000 mg target dose (214/262 81%) were challenged with 6000 mg of peanut protein and all but 1 patient passed the challenge. 14 had demonstrated sustained unresponsiveness with 6000 mg

Legend: Y: years; mo: months; n: number; OFC: oral food challenge; OIT: oral immunotherapy; tot: total; wk: weeks; SLIT sublingual immunotherapy.

**Table 3:** Egg OIT studies.

Reference, Year	Design	Sample Size	Subject Age (yrs)	Maintenance Dose	Duration	Conclusions
**Buchanan AD. et al., 2007** [72]	open-label	7	1–16	300 mg	24 mo	57% passed 8 g OFC. 29% passed OFC after 3–4 mo period of egg avoidance
**Vickery BP. et al., 2010** [73]	open-label	8	3–13	300–3600 mg	18–50 mo	75% passed a 10 g OFC 1 mo after stopping OIT
**Burks AW. et al., 2012** [28]	randomized, placebo controlled	40	5–11	1600 mg	22 mo	75% passed 10 g OFC, but only 28% demonstrated SU on re-challenge 6–8 wk later
**Escudero C. et al., 2015** [74]	double-blind, placebo-controlled	30 (61 to, 30 OIT, 31 controls)	5–17	1 undercooked egg every 48 h	3 mo	At 4 months, 1/31 (3%) in CG passed DBPCFC and 11/30 (37%) of OITG (95% CI, 14 to 51%; *p* = 0.003
**Giavi S. et al., 2016** [75]	double-blind, placebo-controlled	29	1–5.5	9000 mg of low allergenic hydrolyzed egg (HydE) preparation	6 mo	No statistically significant difference was observed on the final OFC (36% and 21% had a negative OFC in the treatment and placebo groups, respectively)
**Yanagida N. et al., 2016** [76]	open-label	21 (33 tot, 21 OIT and 12 controls)	5–18	62 to 194 mg (= 1/32 of a heated whole egg) of egg protein in a scrambled form once daily	12 mo	Respectively, 71% (15/21) and 0% (0/12) of the patients in the OIT and control groups exhibited sustained unresponsiveness to 1/32 of a whole egg 2 weeks after stopping OIT after 12 months (*p* < 0.001); 33% (7/21) and 0% (0/12; *p* = 0.032), respectively, showed sustained unresponsiveness to 1/2 of a whole egg.
**Jones SM. et al., 2016 (follow-up of Burks et al., 2012)** [14]	randomized, placebo-controlled	40 (55 tot, 40 OIT and 15 controls)	5–18	1600 mg	22 mo	Of 40 E-OIT-treated subjects, 20 (50.0%) of 40 demonstrated SU by year 4. SU after E-OIT is enhanced with a longer duration of therapy and increases the likelihood of tolerating unbaked egg in the diet.
**Pérez-Rangel I. et al., 2017** [77]	double-blind, placebo-controlled	15 (33 to, 15 OIT and 14 controls)	5–18	1 undercooked egg every 48 h	5 mo	A total of 32 patients underwent the egg ROIT protocol (ROIT2). Thirty-one children (96.9%) completed the build-up phase, and 30 completed the maintenance phase, with a 93.8% rate of treatment success at five months
**Akaschi M. et al., 2017** [78]	double-blind, placebo-controlled	18 (36 tot, 18 OIT, 18 controls)	3–15	4000 mg of dry egg powder	6 mo	Eight of the 14 (57%) patients in the OIT group passed 4 g of dry egg powder whereas none of the 16 patients in the “eliminate egg” group
**Maeta A. et al., 2018** [79]	open-label	13	3–8	10 LAC, each containing 79–110 mg of egg white protein	4 mo	After the OIT, 7 participants tolerated 2 g of hard-boiled EW. Four participants did not show any improvement in response to OIT.
**Itoh-Nagato N. et al., 2018** [80]	double-blind, placebo-controlled	45	5–15	60 g of cooked egg and 1 g of EWP	The early start group received rush OIT for three months, while the late-start group continued the egg elimination diet (control). In the next stage, both groups received OIT until all participants had finished 12 months of maintenance OIT	The ratio of the participants in whom an increase of the TD was achieved in the first stage was significantly higher in the early-start group (87.0%), than in the late-start group (22.7%).
**Bird JA. et al., 2019** [81]	open-label	13	1–18	3800 mg of BE	2 y	Eight subjects completed 12 months of BE OIT, and seven subjects passed the 3.8 g BE OFC. After an additional year of daily 3.8 g BE ingestion, six subjects were challenged and 5 passed a 6 g LCE OFC. The study suggests that egg-allergic children reactive to BE may be able to undergo BE OIT to accelerate desensitization to LCE.
**Martín-Muñoz MF. et al., 2019** [82]	Double-blind, placebo-controlled	76 (101 tot, 76 OIT and 25 controls)	6–9	3300 g protein (30 mL of PEW) in 38 patients daily, in 38 patients every two days.	12 mo	At T12, 4/25 (16%) of the total control patients passed the PEW DBPCFC vs. 64/76 (84.21%) OIT patients who had reached the target dose or total desensitization. (*p* = 0.000). At T24, 97.43% OIT patients passed the challenge. Daily OIT maintenance achieves better adherence, effectiveness, and safety

Legend: BE: baked egg; EWP: egg white powder; mo: months; LAC: low egg-allergen cookies; OFC: oral food challenge; OIT: oral immunotherapy; tot: total; yrs: years; LCE: light cooked egg; ROIT: rush OIT; SU: sustained unresponsiveness; E-OIT: Egg-OIT; PEW: pasteurized egg white; CG: control group; OITG: OITgroup.

**Table 4:** Tree nut OIT study.

Reference, Year	Design	Samples Size (n)	Subject Age	Maintenance Dose (mg)	Duration	Conclusions
**Elizur A et al., 2019** [83]	randomized, elimination diet controlled	73	4–20 yrs	1200	18 mo	89% desensitized (passed the OFC with 4000 mg of walnut)

Legend: mo: months; n: numbers; yrs: years; OFC: oral food challenge.

**Table 5:** Wheat OIT studies.

Reference, year	Design	Samples Size (n)	Subject Age (yrs)	Maintenance Dose	Duration	Conclusions
**Rodriguez del Rio et al., 2014** [84]	prospective, no control	6	5–11	13 g	6 mo	85% desensitized
**Sato S et al., 2015** [85]	prospective, historical control	29	Median age: 9	1300 mg starting dose Ending dose 5200 mg	24 mo	88.9% desensitized, 61.1% sustained unresponsiveness (passed the OFC with 4000 mg of wheat)
**Okada et al., 2016** [86]	retrospective	57	1–11.8	400 mg	1 yrs	32 patients (86%) tolerated very low dose OFC (53 g of wheat protein)
**Khayatzadeh A et al., 2016** [87]	case-control	13	5.5–19	5.2 g of wheat protein	Build-up phase: 3–6 days; maintenance phase: 3 months	12 out of 13 completed maintenance phase: 12 out of 12 were desensitized
**Rekabi M et al., 2017** [88]	prospective, no control	12	2–10	30–70 g	up-dosing phase: 7.5 months; maintenance dose: 18 months	12 out of 12 patients tolerated 50 g of pasta

Legend: mo: months; n: numbers; yrs: years; OFC: oral food challenge; OIT: oral immunotherapy; Baked Egg: BE.
